# Mitochondrial cardiomyopathy presenting as isolated cardiac involvement with rapid progression due to the MT-TI m.4300A>G mutation

**DOI:** 10.1093/ehjcr/ytaf198

**Published:** 2025-04-18

**Authors:** Lingcheng Zhu, Jinghui Li, Minjie Lu

**Affiliations:** Department of Magnetic Resonance Imaging, Fuwai Hospital and National Center for Cardiovascular Diseases, Chinese Academy of Medical Sciences, Peking Union Medical College, Beilishi Road No.167, Xicheng District, Beijing 100037, China; Department of Radiology, Zibo Central Hospital, No. 54, Gongqingtuan West Road, Zibo 255036, Shandong Province, China; Department of Magnetic Resonance Imaging, Fuwai Hospital and National Center for Cardiovascular Diseases, Chinese Academy of Medical Sciences, Peking Union Medical College, Beilishi Road No.167, Xicheng District, Beijing 100037, China; Department of Magnetic Resonance Imaging, Fuwai Hospital and National Center for Cardiovascular Diseases, Chinese Academy of Medical Sciences, Peking Union Medical College, Beilishi Road No.167, Xicheng District, Beijing 100037, China

**Figure 1:**
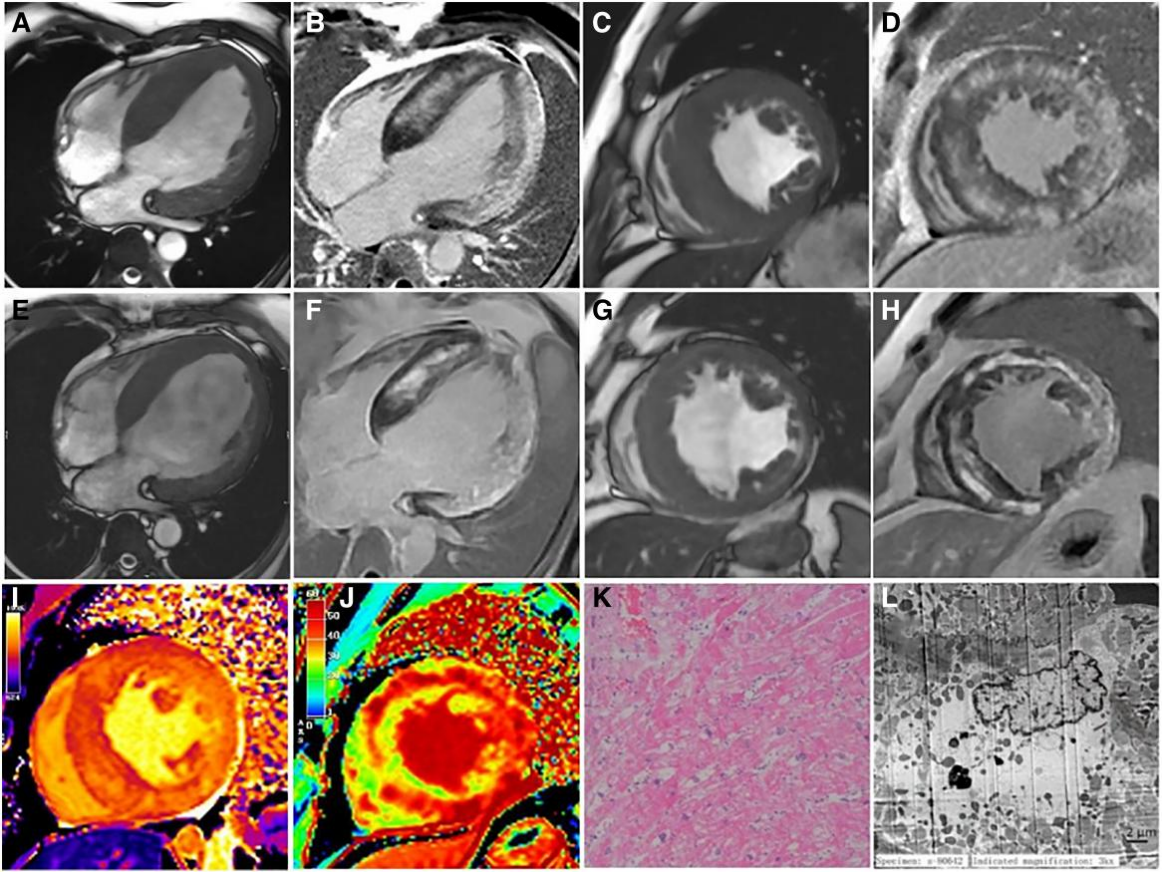
Differential diagnosis of myocardial calcifications and the role of multimodal imaging.

A 35-year-old man presented with chest tightness and shortness of breath for 3 months. He was diagnosed with hypertrophic non-obstructive cardiomyopathy by echocardiography at a local hospital and admitted for further evaluation. Laboratory tests showed elevated N-terminal pro-B-type natriuretic peptide at 655 pg/mL and high-sensitivity cardiac troponin I at 1.787 ng/mL, indicating cardiac stress and injury. Additional evaluations, including dynamic electrocardiogram monitoring and coronary computed tomography angiography, showed sinus arrhythmia and occasional premature ventricular complexes, but no coronary stenosis. Echocardiography demonstrated left ventricular hypertrophy and reduced ejection fraction (45%).

Cardiac magnetic resonance imaging (CMR) provided key insights into the structural abnormalities. Cardiac magnetic resonance imaging revealed diffuse left ventricular wall hypertrophy (interventricular septal thickness: 28 mm, left ventricular diameter: 65 mm) without outflow obstruction, reduced left ventricular function (44%), and extensive subendocardial-midwall late gadolinium enhancement in the interventricular septum and free left ventricular wall (*Panels A–D*, Supplementary data online, Videos first 4ch/sax), indicating infiltrative cardiomyopathy. Initial T1 values and extracellular volume fraction were elevated (*Panels I* and *J*). Three years later, CMR showed increased left ventricular cavity size (diameter: 80 mm), thinner ventricular wall (interventricular septal thickness: 18 mm), and increased fibrosis, indicating progression to dilated cardiomyopathy (*Panels E–H*, Supplementary data online, Videos second 4ch/sax).

A right ventricular endocavitary myocardial biopsy revealed non-specific microstructural changes but no signs of infiltrative cardiomyopathy (*Panels K* and *L*). Complete mitochondrial DNA sequencing identified a homoplasmic pathogenic variant, m.4300A>G, in the mitochondrially encoded tRNA isoleucine (MT-TI) gene, responsible for mitochondrial cardiomyopathy.

The patient is awaiting heart transplant. This case suggests that the MT-TI m.4300A>G mutation can lead to isolated cardiac involvement without systemic manifestations, marked by diffuse myocardial fibrosis and rapid progression. Clinicians encountering similar imaging features should consider testing for mitochondrial tRNA gene mutations.

## Supplementary Material

ytaf198_Supplementary_Data

## Data Availability

The data underlying this article are available from the corresponding author on reasonable request.

## Supplementary material

**Consent**: The authors confirm that written consent has been obtained from the patient for the submission and publication of this article, including images, in accordance with the COPE guidelines.

**Funding**: None declared.

